# Supplementary material for: Proportion of asymptomatic infection among COVID-19 positive persons and their transmission potential: A systematic review and meta-analysis
Source: PLoS One. 2020 Nov 3;15(11):e0241536. doi: 10.1371/journal.pone.0241536 (PMC7608887; doi:10.1371/journal.pone.0241536)
Supplement: S1 File — (DOCX) [file pone.0241536.s002.docx]

# **S1 File**. Data extraction form.

| Author, Year | publication type (peer, pre-print | Country | Population (contacts, travellers, general, etc) | Study design | Setting | Inclusion criteria into study | Study duration period | Follow up time after diagnosis | How many times tested | Time between tests | Definition of asymptomatic | Definition of minimally symptomatic |
| --- | --- | --- | --- | --- | --- | --- | --- | --- | --- | --- | --- | --- |
|  |  |  |  |  |  |  |  |  |  |  |  |  |
| Diagnostic method | Sampling method (nasopgaryngeal, oropharyngeal, etc) | N  eligible to be tested | N  tested | Covid +  (% of tested) | Asympt Covid+ all n (% of all tested ) | Mild Covid+ all n(% of all tested) | asympt+mild all n(%of all tested) | Asymp. Covid + n(% of Covid +) | Mild Covid + n(%of Covid +) | Asympt+mild n(%of Covid +) | Other characteristics | Sex (%male) all |
|  |  |  |  |  |  |  |  |  |  |  |  |  |
| Sex (%male) asympt/mild only | Age estimate (mean median) | Age value for all participants | Age for asympt/mild only | Number of contacts infected (if cluster/contact) | Time to symptoms and severity | Asymp. Covid + became symp. N(%) | Mean time to symptom onset (days) | Range of days to symptom onset | Symptoms developed (with % of each symptom if available) | Mild covid+ became severe n(%) | Mean time to severity (days) | Range of days to severity |
|  |  |  |  |  |  |  |  |  |  |  |  |  |
| Symptom severity (definition of severity) | Time from exposure to testing | Other findings |  |  |  |  |  |  |  |  |  |  |
