# Supplementary material for: Proportion of asymptomatic infection among COVID-19 positive persons and their transmission potential: A systematic review and meta-analysis
Source: PLoS One. 2020 Nov 3;15(11):e0241536. doi: 10.1371/journal.pone.0241536 (PMC7608887; doi:10.1371/journal.pone.0241536)
Supplement: S2 File — (DOCX) [file pone.0241536.s003.docx]

# **S2 File.** Criterial for quality assessment in included studies.

1. Cohort studies

| **1.1 Is the cohort representative of the target population?** | **1.2 Is there likely selection bias?** | **2.1 Was there a clear definition for an asymptomatic case?** | **2.2 Is there likely reporting bias?** | **3.1 Were objective, standard criteria used for diagnosis of the condition?** | **3.2 Was symptom development assessed in asymptomatic subjects?** | **3.3 Was follow-up long enough for symptoms to occur? (14 days on average)** | **3.4 Were symptoms assessed in a systematic and reliable way?** | **3.5 Adequacy of follow up of cohorts (lost to follow up)** | **3.6 Is there likely detection bias?** |
| --- | --- | --- | --- | --- | --- | --- | --- | --- | --- |

Grading scale:

If question 1.2 is answered “yes” then the study is automatically low quality. If one question assessing bias (2.2 or 3.6) is answered "yes" then study is moderate quality. If both questions assessing bias (2.2 and 3.6) are answered "yes" then the study is low quality.

1. Cross- sectional studies

| **1.1 Were the criteria for inclusion in the sample clearly defined?** | **1.2 Was an appropriate method of sampling used? Random, complete, other.** | **1.3 Participation rate** | **1.4 Were the study subjects representative of the target population?** | **1.5 Is there likely to be selection bias?** | **2.1 Was there a clear definition for an asymptomatic case?** | **2.2 Were the study subjects and the setting described in detail?** | **2.3 Is there likely to be reporting bias?** | **3.1 Were objective, standard criteria used for diagnosis of the condition?** | **3.2 Was symptom assessment carried out in a standard objective way?** | **3.3 Is there likely detection bias?** |
| --- | --- | --- | --- | --- | --- | --- | --- | --- | --- | --- |

Grading scale:

If questions 1.2 is not a random sample, or if question 1.3 has a lower than 70% participation rate, then the study is automatically low quality. If one question assessing bias (1.5, 2.3 or 3.3) is answered "yes" then study is moderate quality. If two or more questions assessing bias (1.5, 2.3 or 3.3) are answered "yes" then the study is low quality.

1. Case series

| **1.1 Were the inclusion criteria for the case series clearly defined?** | **1.2 Were valid methods used to diagnose COVID-19 for all participants included in the case series?** | **1.3 Did the case series have consecutive inclusion of participants?** | **1.4 Did the case series have complete inclusion of participants?** | **1.5 Is there likely selection bias ?** | **2.1 Was there clear reporting of the demographics of the participants in the study?** | **2.2 Was there clear reporting of clinical information of the participants?** | **2.3 Were the outcomes or follow up results of cases clearly reported?** | **2.4 Was there clear reporting of the presenting site(s)/clinic(s) demographic information?** | **2.5 Is there likely reporting bias?** |
| --- | --- | --- | --- | --- | --- | --- | --- | --- | --- |

Grading scale:

If question 1.1 is answered "no" then the study is automatically low quality, and if 1.1 is answered "yes" but questions 1.3 or 1.4 are answered "no" then the study is also low quality. If one question assessing bias (1.5 or 2.5) is answered "yes" then study is moderate quality. If both questions assessing bias (1.5 or 2.5) are answered "yes" then the study is low quality.

1. Case series on transmission within clusters

| **1.1 Is the method for contact tracing clearly defined?** | **1.2 Is the number of contacts identified and tested clearly reported?** | **1.3 Is there likely bias due to missing information? i.e reporting bias** | **2.1 Is the method for contact tracing rigorous and exhaustive to identify all potential contacts?** | **2.2 Was the follow-up of people without symptoms sufficient to allow for the incubation period?** | **2.3 Is there likely missing data on contacts?** | **2.4 Is there likely bias in contact identification?** | **2.5 Is there likely exposure to other potential index cases that were symptomatic?** | **2.6 Was the index case symptomatic at any time during the exposure to contacts?** | **2.7 Was the index case identified after the contacts? i.e was the index case identified by exclusion** | **2.8 Is there likely bias in index case identification?** |
| --- | --- | --- | --- | --- | --- | --- | --- | --- | --- | --- |

Grading scale:

If questions 2.6 AND 2.7 are answered "yes" then the study is automatically low quality. If one question assessing bias (1.3, 2.4 or 2.8) is answered "yes" then study is moderate quality. If two or more signalling questions are answered “yes” then the study is low quality. For studies on household transmission, signalling questions 1.1 and 1.2 were omitted.
