# Supplementary material for: Proportion of asymptomatic infection among COVID-19 positive persons and their transmission potential: A systematic review and meta-analysis
Source: PLoS One. 2020 Nov 3;15(11):e0241536. doi: 10.1371/journal.pone.0241536 (PMC7608887; doi:10.1371/journal.pone.0241536)
Supplement: S1 Table — (DOCX) [file pone.0241536.s004.docx]

# **S1 Table**. Quality assessment of all included studies.

| **Author, Country** | **Type of study** | **Publication type** | **Quality** | **Domains with Potential Bias Concerns** |
| --- | --- | --- | --- | --- |
| Aarons, M. USA | Cohort | Peer reviewed | Moderate | Detection |
| Albalate, M. Spain | Cohort | Peer reviewed | Low | Selection, Reporting, Detection |
| Al-Shamsi, H. UAE | Cohort | Peer reviewed | Low | Selection |
| Andrikopolou, M. USA | Cohort | Peer reviewed | High | None |
| Arima, Y. Japan | Cohort | Peer reviewed | Low | Reporting, Detection |
| Baggett, T. USA | Cross sectional | Peer reviewed | Moderate | Reporting |
| Bianco, A. USA | Cross sectional | Peer reviewed | High | None |
| Brandstetter, S. Germany | Cross sectional | Peer reviewed | Low | Selection, Reporting |
| Breslin, N. USA | Cohort | Peer reviewed | Low | Reporting, Detection |
| Brown, C. UK | Cross sectional | Pre-print | Low | Selection, Reporting |
| Chamie, G. USA | Cohort | Pre-print | Low | Selection, Reporting |
| Chaw, L. Brunei | Cohort | Pre-print | Low | Selection, Detection |
| Cohen, R. France | Cross sectional | Pre-print | Low | Selection, Reporting |
| Dora, A. USA | Cohort | Peer reviewed | Moderate | Detection |
| Doung-nern, P. Thailand | Cross sectional | Pre-print | Low | Reporting, Detection |
| Freyburg, A. Germany | Cross sectional | Peer reviewed | Low | Reporting, Detection |
| Goldfarb, I. USA | Cohort | Peer reviewed | Moderate | Detection |
| Graham, N. UK | Cohort | Peer reviewed | Moderate | Detection |
| Gruskay, J. USA | Cohort | Peer reviewed | Low | Selection, Detection |
| Gudbjartsson, D. Iceland | Cross sectional | Peer reviewed | Low | Selection |
| Hung, I. Hong Kong | Cohort | Peer reviewed | Low | Selection |
| Ing, A. Australia | Cohort | Peer reviewed | Low | Selection, Reporting, Detection |
| Jatt, L. USA | Cohort | Peer reviewed | Low | Selection, Reporting, Detection |
| Khalil, A. UK | Cohort | Pre-print | Low | Reporting, Detection |
| Kraehling, V. Germany | Cohort | Pre-print | Low | Selection, Reporting, Detection |
| Kimball, A. USA | Cohort | Peer reviewed | Moderate | Detection |
| LaCourse, S. USA | Cohort | Pre-print | Low | Reporting, Detection |
| Lai, X. China | Cross sectional | Peer reviewed | High | None |
| Lan, F. USA | Cross sectional | Pre-print | Moderate | Reporting |
| Lavezzo, E. Italy | Cohort | Peer | Moderate | Detection |
| Lombardi, A. Italy | Cohort | Peer | High | None |
| London, V. USA | Cohort | Peer reviewed | Moderate | Detection |
| Luo, L. China | Cohort | Pre-print | Low | Selection, Detection |
| Ly, T. France | Cross sectional | Pre-print | High | None |
| Lytras, T. Greece | Cohort | Peer reviewed | Low | Selection, Reporting |
| Neishiura, H. Japan | Cohort | Peer reviewed | Low | Reporting, Detection |
| Ochiai, D. Japan | Cohort | Peer reviewed | Moderate | Detection |
| Olalla, J. Spain | Cross sectional | Pre-print | Low | Selection |
| Ossami, R. Germany | Cohort | Peer reviewed | High | None |
| Park, S. South Korea | Cohort | Peer reviewed | High | None |
| Patel, M. USA | Cohort | Pre-print | High | None |
| Romao, VC. Portugal | Cohort | Pre-print | Moderate | Reporting |
| Roxby, A. USA | Cohort | Peer reviewed | Moderate | Reporting |
| Samuels, E. USA | Cross sectional | Pre-print | Low | Selection, Reporting |
| Schwierzeck, V. Germany | Cohort | Peer reviewed | Moderate | Detection |
| Snoeck, C. Luxemburg | Cohort | Pre-print | Moderate | Reporting |
| Son, H. South Korea | Cohort | Pre-print | Low | Selection |
| Sutton, D. USA | Cohort | Peer reviewed | Moderate | Reporting |
| Tabata, S. Japan | Cohort | Peer reviewed | Low | Selection |
| Tian, S. China | Cohort (retrospective) | Pre-print | Low | Selection, Reporting, Detection |
| Yin, G. China | Cohort | Peer | Low | Selection, Reporting, Detection |
| Zhang, J. China | Cross sectional | Peer reviewed | Moderate | Detection |
| Studies on transmission | | | | |
| Bai, Y. China | Transmission cluster | Peer | Low | Reporting, contact identification and index case identification |
| Chen,W. China | Transmission cluster | Peer | Low | Reporting, contact identification |
| Cheng, H. Taiwan | Transmission cluster | Peer | Low | Reporting, contact identification |
| Hijnen, D. Germany | Transmission cluster | Peer | Low | Reporting, contact identification |
| Hu, Z. China | Transmission cluster | Peer | Low | Reporting, contact identification and index case identification |
| Huang, L. China | Transmission cluster | Peer | High | None |
| Jiang, X. China | Transmission cluster | Pre-print | Low | Contact identification and index case identification |
| Li, C. China | Transmission cluster | Peer | Low | Reporting, contact identification and index case identification |
| Li, P. China | Transmission cluster | Peer | Moderate | Reporting |
| Lu, S. China | Transmission cluster | Peer | Low | Reporting, contact identification and index case identification |
| Luo, S. China | Transmission cluster | Peer | Low | Reporting, contact identification and index case identification |
| Qian, G. China | Transmission cluster | Peer | Low | Reporting, contact identification |
| Qiu, Ch. China | Transmission cluster | Pre-print | Low | Reporting, contact identification and index case identification |
| Rothe, C. Germany | Transmission cluster | Peer | Low | Reporting, contact identification |
| Tong, Z. China | Transmission cluster | Peer | Low | Reporting, contact identification |
| Xiao, W. China | Transmission cluster | Peer | Moderate | Reporting |
| Ye, F. China | Transmission cluster | Peer | Moderate | Reporting |
| Yu, P. China | Transmission cluster | Peer | Low | Reporting, contact identification |
| Zhang, J. China | Transmission cluster | Peer | Low | Reporting, contact identification and index case identification |
