# Supplementary material for: Proportion of asymptomatic infection among COVID-19 positive persons and their transmission potential: A systematic review and meta-analysis
Source: PLoS One. 2020 Nov 3;15(11):e0241536. doi: 10.1371/journal.pone.0241536 (PMC7608887; doi:10.1371/journal.pone.0241536)
Supplement: S2 Table — (DOCX) [file pone.0241536.s005.docx]

# **S2 Table**. Low quality studies reporting population characteristics, COVID-19 prevalence, and proportion of asymptomatic infection among COVID-19 positive persons at time of testing.

| **Author, Country** | **Male Sex**  **(%)** | **Age**  **(Years)** | **Percent of eligible population who were tested** | **COVID-19 positive and number tested**  **n/N (%)** | **Proportion of asymptomatic infection at initial testing for COVID-19**  **%** |
| --- | --- | --- | --- | --- | --- |
| General Population Studies |  |  |  |  |  |
| Gudbjartsson, D.  Iceland  (Random sample of population)^2^ | COVID-19 Positive:  69.2% | Mean^1^:  50.5 | 34% | 13/2283 (0.6) | 54% |
| Gudbjartsson, D.  Iceland  (Self-selected population)^2^ | COVID-19 Positive: 52.9% | Mean^1^:  40.8 | NA | 87/10 797 (0.8) | 41% |
| Chamie, G.  USA^*^ | COVID-19 positive:  76% | Median (IQR)^1^:  38 (28-50) | 74.8% | 83/3871 (2.1%) | 39.8%^3^ |
| Khraling, V.  Germany* | Tested population:  78.5% | NA | 100% | 1/1000 (0.1%) | 100% |
| Son, H.  South Korea* | COVID-19 positive:  45.4% | NA | 100% | 108/18 303 (0.6%) | 11.1% |
| Studies in contacts |  |  |  |  |  |
| Chen, Y.  China | NA | NA | NA | 110/2147 (5.1%) | 20% |
| Doung-nern, P.  Thailand* | COVID-19 positive:  69% | Distribution:  46% between 40 and 65 | 88.6% | 211/930 (22.7%) | 28.9% |
| Luo, L.  China* | Tested Population: 50.2% | Median (IQR):  38 (25-52) | 94.0% | 129/4653 (2.8%) | NA |
| Tian, S.  China* | COVID-19 Positive:  37.5% | Mean (SD)^1^:  48 (1.7) | NA | 24/~8000 (0.3%)^4^ | 29.2% |
| Yin, G.  China* | NA | NA | 95.5% | 100/2050 (4.9%) | 18.0% |
| Other Population Studies |  |  |  |  |  |
| *Health Care Workers in Settings Other than Nursing Homes* |  |  |  |  |  |
| Brandstetter, S.  Germany | COVID-19 positive:  16.1% | Distribution^1^:  46.7% between 18 and 35 | 53.0% | 31/201 (15.4%) | 3.2% |
| Freyburg, A.  Germany | NA | NA | 100% | 58/1170 (5.0%) | 75.9% |
| Brown, C.  UK^*^ | COVID-19 positive:  34.8% | Median (IQR):  39 (19-68) | NA | 23/1149 (2.0%) | 17.4% |
| Olalla, J.  Spain* | Tested Population:  30% | Mean:  41.5 | NA | 2/498 (0.4%) | 50% |
| *Obstetric Patients Presenting to Hospitals* |  |  |  |  |  |
| Breslin, N.  USA | COVID-19 positive:  0% | Mean (SD)^1^:  26.9 (5.9) | NA | 36^5^ | 33.3% |
| Khalil, A.  UK* | COVID-19 positive:  0% | Median^1^:  34 | 100% | 9/129 (7.0%) | 88.9% |
| LaCourse, S.  USA* | COVID-19 positive:  0% | NA | 100% | 5/188 (2.7%) | 20% |
| *Congregate settings* |  |  |  |  |  |
| Samuels, E.  USA* | COVID-19 positive:  74% | Distribution^1^:  66% between 40 and 64 | NA | 35/299 (11.7%) | 80% |
| *Nursing Homes* |  |  |  |  |  |
| Jatt, L.  USA | Tested population:  77% | Mean (SD):  68.5 (15) | 100% | 18/149 (12.1%) | 88.9% |
| *Travelers* |  |  |  |  |  |
| Arima, Y. & Neishiura, H.  Japan | Tested Population:  89% | NA | 100% | 12/566 (2.1%) | 41.6% |
| Chaw, L.  Brunei* | NA | NA | 100% | 19/75 (25.3%) | 57.9% |
| Hung, I.  Hong Kong | COVID-19 positive:  33% | Median (IQR)^1^:  58 (56-61) | 100% | 8/215 (3.7%) | 62.5% |
| Ing, A.  Australia | NA | NA | 100% | 128/217 (59%) | 81% |
| Lytras, T.  Greece | NA | Median (IQR):  27 (22-40) | 100% | 40/783 (5.1) | 97.5% |
| Tabata, S.  Japan | COVID-19 positive:  44% | Median (IQR)^1^:  69 (61-75) | 100% | NA | 41.3% |
| *Hemodialysis Patients* |  |  |  |  |  |
| Albalate, M.  Spain | COVID-19 positive:  62.1% | NA | 100% | 36/90 (40%) | 41.7% |
| *Cancer Patients* |  |  |  |  |  |
| Al-Shamsi, H.  UAE | Tested Population:  43.5% | Median (IQR):  55 (28-76) | 100% | 7/85 (8.2%) | 100% |
| *Patients Admitted to Hospitals for Orthopedic Surgery* |  |  |  |  |  |
| Gruskay, J.  USA | COVID-19 positive:  50% | Mean (SD)^1^:  66.0 (27.2) | 70.2% | 12/99 (12.1%) | 58.3% |
| *Pediatric Studies* |  |  |  |  |  |
| Cohen, R.  France* | Tested population:  53.2% | Mean (SD):  4.9 (3.9) | 100% | 11/605 (1.8%) | 36.4% |

Abbreviations: IQR: Interquartile range; SD: Standard Deviation ; NA: Not available in the paper; *Pre-print studies.

Notes:

1) Age among COVID-19 positive patients only.

2) In Gudbjartsson, D. et al. two different populations were tested (random sample and self-selected), these were considered as two cohorts.

3) In Chamie, G. et al., there were 43 asymptomatic persons at the time of testing. 41 persons were followed up and 8 of those recalled being symptomatic prior to initial testing and 33 truly asymptomatic at the initial test.

4) In Luo, L. et al., the denominator is reported as “about 8000”

5) In Breslin, N. et al., there is no information on the total normal of women tested. 43 women in total tested COVID-19 positive, 36 of which were tested during the universal screening portion of the study.
