# Supplementary material for: Proportion of asymptomatic infection among COVID-19 positive persons and their transmission potential: A systematic review and meta-analysis
Source: PLoS One. 2020 Nov 3;15(11):e0241536. doi: 10.1371/journal.pone.0241536 (PMC7608887; doi:10.1371/journal.pone.0241536)
Supplement: S3 Table — (DOCX) [file pone.0241536.s006.docx]

# **S3 Table**. Low quality studies reporting proportion of asymptomatic infections among COVID-19 positive persons through follow-up, and time to symptom onset among those developing symptoms in follow-up.

| **Author, Country^2^** | **Proportion of asymptomatic infection among COVID-19 positive persons at initial testing**  **% (n/N)** | **Proportion of COVID-19 infection that is asymptomatic throughout follow-up**  **% (n/N)** | **Follow up time after initial testing (days)** | **Days to symptom onset among those asymptomatic at testing and who developed symptoms during follow up** |
| --- | --- | --- | --- | --- |
| **General Population** |  |  |  |  |
| Chamie, G.  USA | 39.8% (33/83)^3^ | 27.7% (23/83) | For all:  14 days | NA |
| Khraling, V.  Germany | 100% (1/1) | 0% (0/1) | NA | For all:  5 days |
| Son, H.  South Korea | 11.1% (12/108) | 3.7% (4/108) | NA | NA |
| **Contacts** |  |  |  |  |
| Luo, L.  China | NA | 6.2% (8/129) | Median (Range):  4 (3 to13) | NA |
| **Healthcare workers in settings other than nursing homes** |  |  |  |  |
| Brown, C.  UK | 17.4% (4/23) | 0% (0/23)^4^ | For all:  7 days | NA |
| **Obstetric Patients Presenting to Hospitals** |  |  |  |  |
| Breslin, N.  USA | 33% (12/36) | 16.7% (6/36) | For all:  14 days | Mean:  7 days |
| LaCourse, S.  USA | 20% (1/5) | 20% (1/5) | Up to 18 days |  |
| **Travelers** |  |  |  |  |
| Hung, I.  Hong Kong | 62.5% (5/8) | 50% (4/8) | For all:  14 days | For all:  2 days |
| Arima, Y. & Neishiura, H.  Japan | 41.6% (5/12) | 25% (3/12) | Range:  14 to 30 days | Range:  2 to 4 days |
| Lytras, T.  Greece | 97.5% (39/40) | 87.5% (35/40) | Range:  13 to 18 | NA |
| Tabata, S.  Japan | 41.3% (43/104)^5^ | 31.7% (33/104) | Range:  3 to 15 days | NA |
| Chaw, L.  Brunei * | 57.9% (11/19) | 21.1% (4/19) | NA | NA |
| **Hemodialysis Patients** |  |  |  |  |
| Albalate, M.  Spain | 41.7% (15/36) | 38.9% (14/36)^6^ | NA | 2 days^6^ |
| **Cancer Patients** |  |  |  |  |
| Al-Shamsi, H.  UAE | 7/7 (100%) | 0% (0/7) | Range:  26 to 47 days | NA |
| **Patients Admitted to Hospitals for Orthopedic Surgery** |  |  |  |  |
| Gruskay, J.  USA | 58.3% (7/12) | 33.3% (4/12) | NA | NA |

Abbreviations: IQR: Interquartile range; NA: Not available in the paper. * Pre-print studies

Notes:

1) Studies with repeated testing were considered as only one cohort.

2) No studies were blinded to COVID-19 diagnosis. Time between COVID-19 exposure to initial test was not available.

3) In Chamie, G. et al., there were 43 asymptomatic persons at the time of testing. 41 persons were followed up and 8 of those recalled being symptomatic prior to initial testing and 33 truly asymptomatic at the initial test.

4) In Brown, C. et al., the text states that no asymptomatic persons developed symptoms, but their Supplementary table (S1) reports one person who developed symptoms.

5) In Tabata, S. et al., it is not stated how many people were COVID-19 positive in the total tested population. 104 COVID-19 positive persons were included in this study.
