# Supplementary material for: Proportion of asymptomatic infection among COVID-19 positive persons and their transmission potential: A systematic review and meta-analysis
Source: PLoS One. 2020 Nov 3;15(11):e0241536. doi: 10.1371/journal.pone.0241536 (PMC7608887; doi:10.1371/journal.pone.0241536)
Supplement: S4 Table — (DOCX) [file pone.0241536.s007.docx]

# **S4 Table**. Transmissibility of infection for asymptomatic and pre-symptomatic patients: from studies on contact investigations

| **Author** | **Exposure setting** | **Quality assessment** | **N of index patients** | **Age of index patient in years (mean, SD)** | **N of contacts of index patients** | **N subsequently infected contacts** | **Mean days from first exposure to symptom onset in index patient** | **Mean days from first exposure to symptom onset in positive contacts** | **Mean days from first exposure to testing in positive contacts** | **Attack rate, %** |
| --- | --- | --- | --- | --- | --- | --- | --- | --- | --- | --- |
| **Studies in Asymptomatic Index Patients** | | | | | | | | | | |
| Park S, Apr 2020(1) | Family cluster | High | 4 | NR | 4 | 0 | -- | -- | 5‡ | 0 |
| Bai, Y. Feb 2020 (2) | Family cluster | Low | 1 | 20 | 5 | 5 | -- | 11.4 | 14.6 | 100 |
| Cheng H, May 2020 (3) | Close contacts | Low | 9 | NR | 91 | 0 | -- | -- | NR | 0 |
| Hu Z, Mar 2020 (4) | Family cluster | Low | 1 | 67 | 3 | 3 | -- | 7 | 13 | 100 |
| Qiu, C. Mar 2020 (5) | Family cluster | Low | 2 | NR | 3 | 3 | -- | NR | 16 | 100 |
| Luo, S. 2020 (6) | Family cluster | Low | 1 | 50 | 1 | 1 | -- | 20 | 19 | 100 |
| Zhang, J. Mar 2020 (7) | Close contacts | Low | 1 | 48 | 3 | 2 | -- | 6 | 8.5 | 66.6 |
| **Studies in Pre-symptomatic Index Patients*** | | | | | | | | | | |
| Huang, L. Mar 2020 (8) | Close contacts | High | 1 | 22 | 22 | 7 | 4 | 2 | 6.2 | 31.8 |
| Park S, Apr 2020 (1) | Family cluster | High | 4 | NR | 11 | 0 | -- | Asymptomatic | 5‡ | 0 |
| Li, P. Mar 2020 (9) | Family cluster | Moderate | 1 | 51** | 5 | 4 | 13 | 10 | 10 | 80 |
| Ye, F. Mar 2020 (10) | Close contacts | Moderate | 1 | 50 | 44 | 4 | 5 | 10.6 | 9 | 9.1 |
| Xiao, W. 2020 (11) | Close contacts | Moderate | 2 | NR | 10 | 3 | -- | NR | 4.6 | 33.3 |
| Chen W, 2020 (12) | Family cluster | Low | 2 | 64 and 58 | 4 | 4 | 5 | 7 | 9.75 | 100 |
| Chen W, 2020 (12) | Family cluster | Low | 1 | NR | 6 | 6 | 7 | 4.8 | 9 | 100 |
| Hijnen D, 2020 (13) | Close contacts | Low | 1 | 57 | 11 | 10 | 4 | 4.2 | 8.4 | 91 |
| Jiang, X. Apr 2020 (14) | Family cluster | Low | 2 | 37 and 35 | 9 | 4 | 10 | 14 | 11.3 | 44.4 |
| Li C. Mar 2020 (15) | Family cluster | Low | 1 | 56 | 5 | 5 | -- | 10.8 | 10.1 | 100 |
| Lu, S. 2020 (16) | Family cluster | Low | 1 | NR | 2 | 2 | 14 | Asymptomatic | 18 | 100 |
| Qian, G. Mar 2020 (17) | Family cluster | Low | 2 | 58 and 60 | 4 | 3 | 5 | 11 | 12 | 75 |
| Qian, G. Mar 2020 (17) | Family cluster | Low | 3 | 13 to 32 | 3 | 3 | 5 | 10.6 | 12.3 | 100 |
| Rothe, C. Mar 2020 (18) | Work cluster | Low | 1 | NR | 2 | 2 | 4 | 4.5 | 8 | 100 |
| Rothe, C. Mar 2020 (18) | Work cluster | Low | 1 | 33 | 2 | 2 | 5 | 6.5 | 8.5 | 100 |
| Tong, Z. 2020 (19) | family cluster | Low | 1 | 45 | 2 | 2 | 5 | 7 | 16 | 100 |
| Yu, P. Feb 2020 (20) | Family cluster | Low | 2 | 65 and 69 | 2 | 2 | 7.5 | 7.5 | 8 | 100 |

* The index case developed symptoms eventually but did transmit the infection while pre-symptomatic

** Age is reported twice 51 and 56 in Figure 1. † Average for all contacts. ‡ All contacts tested within 5 days of exposure

**References supplementary table 4:**

1. Park SY, Kim YM, Yi S, Lee S, Na BJ, Kim CB, Kim JI, Kim HS, Kim YB, Park Y, Huh IS. Early Release-Coronavirus Disease Outbreak in Call Center, South Korea-Volume 26, Number 8—August 2020-Emerging Infectious Diseases journal-CDC.

2. Bai Y, Yao L, Wei T, Tian F, Jin DY, Chen L, Wang M. Presumed asymptomatic carrier transmission of COVID-19. Jama. 2020 Apr 14;323(14):1406-7.

3. Cheng HY, Jian SW, Liu DP, Ng TC, Huang WT, Lin HH. Contact tracing assessment of COVID-19 transmission dynamics in Taiwan and risk at different exposure periods before and after symptom onset. JAMA internal medicine. 2020 May 1.

4. Hu Z, Song C, Xu C, Jin G, Chen Y, Xu X, Ma H, Chen W, Lin Y, Zheng Y, Wang J. Clinical characteristics of 24 asymptomatic infections with COVID-19 screened among close contacts in Nanjing, China. Science China Life Sciences. 2020 May;63(5):706-11.

5. Qiu C, Deng Z, Xiao Q, Shu Y, Deng Y, Wang H, Liao X, Liu H, Zhou D, Zhao X, Zhou J. Transmission and clinical characteristics of coronavirus disease 2019 in 104 outside‐Wuhan patients, China. Journal of Medical Virology. 2020 Jan 1.

6. Luo SH, Liu W, Liu ZJ, Zheng XY, Hong CX, Liu ZR, Liu J, Weng JP. A confirmed asymptomatic carrier of 2019 novel coronavirus. Chinese medical journal. 2020 May 5;133(9):1123-5.

7. Zhang J, Tian S, Lou J, Chen Y. Familial cluster of COVID-19 infection from an asymptomatic. Critical care. 2020;24(1):119.

8. Huang L, Zhang X, Zhang X, Wei Z, Zhang L, Xu J, et al. Rapid asymptomatic transmission of COVID-19 during the incubation period demonstrating strong infectivity in a cluster of youngsters aged 16-23 years outside Wuhan and characteristics of young patients with COVID-19: a prospective contact-tracing study. 2020.

9. Li P, Fu JB, Li KF, Liu JN, Wang HL, Liu LJ, et al. Transmission of COVID-19 in the terminal stages of the incubation period: A familial cluster. International journal of infectious diseases. 2020;96:452-3.

10. Ye F, Xu S, Rong Z, Xu R, Liu X, Deng P, et al. Delivery of infection from asymptomatic carriers of COVID-19 in a familial cluster. International journal of infectious diseases. 2020;94:133-8.

11. Xiao WJ, Gao Q, Jin K, Gong XH, Han RB, Jiang CY, et al. [Investigation of an epidemic cluster caused by COVID⁃19 cases in incubation period in Shanghai]. Zhonghua liu xing bing xue za zhi = Zhonghua liuxingbingxue zazhi. 2020;41(0):E033.

12. Chen W, Hu C, Huang L, Hu Z, Zeng Y, Wang W, et al. Characterization of a big family cluster infection associated with SARS-Cov-2 in Nanjing district. 2020.

13. Hijnen D, Marzano AV, Eyerich K, GeurtsvanKessel C, Giménez-Arnau AM, Joly P, et al. SARS-CoV-2 Transmission from Presymptomatic Meeting Attendee, Germany. Emerging Infectious Disease journal. 2020;26(8):1935.

14. Jiang XL, Zhang XL, Zhao XN, Li CB, Lei J, Kou ZQ, Sun WK, Hang Y, Gao F, Ji SX, Lin CF. Transmission potential of asymptomatic and paucisymptomatic SARS-CoV-2 infections: a three-family cluster study in China. The Journal of Infectious Diseases. 2020 Apr 22.

15. Li C, Ji F, Wang L, Wang L, Hao J, Dai M, Liu Y, Pan X, Fu J, Li L, Yang G. Asymptomatic and human-to-human transmission of SARS-CoV-2 in a 2-family cluster, Xuzhou, China. Emerging infectious diseases. 2020 Jul;26(7):1626

16. Lu S, Lin J, Zhang Z, Xiao L, Jiang Z, Chen J, Hu C, Luo S. Alert for non‐respiratory symptoms of Coronavirus Disease 2019 (COVID‐19) patients in epidemic period: a case report of familial cluster with three asymptomatic COVID‐19 patients. Journal of medical virology. 2020 Mar 19.

17. Qian G, Yang N, Ma AHY, Wang L, Li G, Chen X, et al. COVID-19 Transmission Within a Family Cluster by Presymptomatic Carriers in China. Clinical infectious diseases.2020;71(15):861-2.

18. Rothe C, Schunk M, Sothmann P, Bretzel G, Froeschl G, Wallrauch C, Zimmer T, Thiel V, Janke C, Guggemos W, Seilmaier M. Transmission of 2019-nCoV infection from an asymptomatic contact in Germany. New England Journal of Medicine. 2020 Mar 5;382(10):970-1.

19. Tong ZD, Tang A, Li KF, Li P, Wang HL, Yi JP, et al. Potential Presymptomatic Transmission of SARS-CoV-2, Zhejiang Province, China, 2020. Emerging infectious diseases. 2020;26(5):1052-4.

20. Yu P, Zhu J, Zhang Z, Han Y. A Familial Cluster of Infection Associated With the 2019 Novel Coronavirus Indicating Possible Person-to-Person Transmission During the Incubation Period. The Journal of Infectious Diseases. 2020;221(11):1757-61.
