# Supplementary material for: Proportion of asymptomatic infection among COVID-19 positive persons and their transmission potential: A systematic review and meta-analysis
Source: PLoS One. 2020 Nov 3;15(11):e0241536. doi: 10.1371/journal.pone.0241536 (PMC7608887; doi:10.1371/journal.pone.0241536)
Supplement: S5 Table — All index patients were asymptomatic when they were in contact with others. (DOCX) [file pone.0241536.s008.docx]

# **S5 Table**. Transmission from asymptomatic/pre-symptomatic index patients to contacts and time to symptoms development in positive contacts (high and moderate quality studies). All index patients were asymptomatic when they were in contact with others.

|  | **Number of Studies** | **Number of Index Patients** | **Contacts** | | **In positive contacts, mean (SD)* days from first exposure to:** | |
| --- | --- | --- | --- | --- | --- | --- |
|  |  |  | **Number of Contacts Tested** | **Number of Contacts Testing Positive** | **Symptom Onset** | **COVID-19 Testing** |
| **Moderate and High Quality Studies** |  |  |  |  |  |  |
| All Contacts | 5 | 13 | 96 | 18 | Reported in 3 studies:  7 (4.6) | Reported in 5 studies:  7 (2.4) |

Notes:

* Means are for entire study cohort.
