# Supplementary material for: Proportion of asymptomatic infection among COVID-19 positive persons and their transmission potential: A systematic review and meta-analysis
Source: PLoS One. 2020 Nov 3;15(11):e0241536. doi: 10.1371/journal.pone.0241536 (PMC7608887; doi:10.1371/journal.pone.0241536)
Supplement: Attachment — Submitted filename: Response to reviewers.docx [file pone.0241536.s009.docx]

October 16, 2020

PLOS ONE

Dear Dr. Serra,

Many thanks to you and the reviewers for your time in reviewing our manuscript. We have revised the paper in light of the comments received and provided a point-by-point response, where necessary. We have provided a track changes and a clean version of the manuscript. In our responses below, when we refer to line numbers, these refer to line numbers in the clean version. We appreciate the opportunity to revise our manuscript.

We look forward to hearing from you.

Yours very truly,

Dick Menzies and Mercedes Yanes Lane, On behalf of all authors

**Editor comments:**

1. Please ensure that your manuscript meets PLOS ONE's style requirements, including those for file naming.

Response: We followed the formatting guidelines and updates as necessary.

2. In the Discussion section, please discuss how results can be interpreted given the quality of the included studies.

Response: This is a helpful comment. We have added the following text to the discussion “We excluded studies assessed to be of low quality, as these did not include information on the population tested, methods for ascertaining the presence of symptoms or definition of asymptomatic”, which we deemed to be important for reducing bias. By including studies that had rigorous methodologies as well as complete reporting, we were able to provide more accurate estimates, however, considerations need to be taken regarding the generalizability of results.” This revision can be found on lines 323 to 328.

3. Please include captions for your Supporting Information files at the end of your manuscript, and update any in-text citations to match accordingly.

Response: We have added captions for the Supporting Information to the end of the manuscript.

**Reviewer comments:**

1. Since the authors mention in the Discussion that data were inadequate to draw conclusions regarding asymptomatic infection and age, a sentence or two in the Introduction summarizing the current state of knowledge regarding symptoms by age would be appropriate.

Response: The following statement (with the appropriate references) has been added to the introduction “observational studies have found that younger patients are less likely to present with severe forms of the disease.” This revision can be found on lines 49 to 50.

2. Line 218 - Superscript the "2" in I2.

Response: We have corrected the superscript.

3. Line 301 - I believe that "COVID-19" should be replaced with "SARS-CoV-2" since the virus is being transmitted, rather than the disease.

Response: Thank you for flagging this. We have changed accordingly.

4. Line 309 - the list of high risk populations that require asymptomatic screening could also include younger people in congregate settings where safety precautions are not strictly followed (i.e., college students).

Response: This is a helpful suggestion. We have updated the list to include populations in congregate settings.
